# Deltamethrin Microencapsulation in Emulsion Paint Binder and Its Long-Term Efficacy Against Dengue Vector *Aedes aegypti*

**DOI:** 10.3389/fpubh.2021.686122

**Published:** 2021-10-25

**Authors:** B. N. Acharya, Rajkumar Ahirwar, Sunil Dhiman, Kavita Yadav, Pratibha Pandey, Devanathan Sukumaran

**Affiliations:** ^1^Synthetic Chemistry Division, Defence R&D Establishment, Gwalior, India; ^2^Vector Management Division, Defence R&D Establishment, Gwalior, India; ^3^Electron Microscopy Division, Defence R&D Establishment, Gwalior, India

**Keywords:** binder, emulsion, deltamethrin, insecticidal paint, dengue vector, *Aedes aegypti*

## Abstract

Various control interventions have been effective in the control of arthropod vectors to a certain extent; still, sustained vector control is an existing problem globally. Insecticide-based formulations have been found to be useful, however the proper delivery of active molecules to target vectors is important. Currently, synthetic pyrethroid deltamethrin (DM) has been microencapsulated in the emulsion paint binder and evaluated for long-term effectiveness against dengue vector *Aedes aegypti*. Different compositions of emulsion binder were prepared by varying the content of monomer and DM. A selection was made for the composition yielding the best combination of properties like solid content, intrinsic viscosity, and DM content. Developed formulation was tested against laboratory-reared and pathogen-free *Ae. aegypti* mosquitoes. Encapsulation of DM in emulsion binder during polymerization showed a uniform distribution. The optimized formulation was stable and did not have a considerable plasticizing effect. Scanning electron microscopy revealed that grain-like micro crystals of DM and surfactant sodium lauryl sulfate (SDS) were uniformly distributed on the formulation surface. The best optimized formulation was highly effective against dengue vector *Ae. aegypti* and found to provide efficacy for up to 18 months of application. The knockdown time (KDT) values KDT_10_ and KDT_50_ were 7.4 min (95% CI: 5.6–9.1) and 22.1 min (95% CI: 19.7–24.3) respectively, whereas 24 h corrected mortality was 90% (95% CI: 82.5–97.5) after 18 months of application (T18). The probit model used to determine knockdown values did not deviate from the linearity and displayed normal distribution of knockdown % with time for different formulations (*p* ≥ 0.1). Presently developed DM microencapsulated emulsion binder was stable, smooth, and uniform. The binder displayed excellent anti-insect property and was capable of providing long-term effectiveness against dengue vectors *Ae. aegypti*. Such a formulation after field-scale evaluation could be very useful in attaining long-term protection from arthropod vectors.

## Introduction

Vector control is an outstanding problem in countries situated in tropical and sub-tropical climate zones. Arthropods such as mosquitoes, houseflies, cockroaches, and ticks spread life-threatening diseases in humans. Insecticides are effective tools for arthropod vectors control with appropriate deployment methods taking into consideration the ambient condition, especially home dwellings, hospitals, food production units, etc. The majority of vector insects rest and crawl on walls and surfaces of a building. Insecticidal paints kill insects usually by contact, therefore coating the interior and external walls and surfaces of civil constructions with insecticide paint could be a cost-effective intervention to control hematophagous insects and nuisance pests. Research on architectural paints containing insecticides, used to coat walls and ceilings of buildings, has gained attention during recent years. The development of novel technology of paint formulations in which microencapsulated insecticides in the form of active ingredients (AIs) have been embedded in the paint matrix has been found to be durable and effective against mosquitoes for a considerably long period of time (1, 2).

However, there are certain drawbacks in the existing insecticidal paint formulations and paints additives. The majority of such products are organic solvent-based and hence not very environmentally friendly (3). This is largely because insecticides are based on chemicals with carbon as the basis of their molecular structure, and therefore may not readily dissolve in aqueous formulations (4). The Weatherall Company Inc. has disclosed a dispersion (BugJuice^®^) containing 4.75% of the insecticide deltamethrin (DM), which can be added to any oil- or latex-based paint. However, once added, the paint must be used within 3 h otherwise the insecticide becomes ineffective. Furthermore, the users of architectural paints prefer not to mix the additives to paint as it is often difficult to achieve a homogenous mixture and sustained release over a long period of time. The process of making insecticide dispersion is complicated and the dispersion itself will contain surfactants that may adversely affect the film properties of the coating.

Waterborne paints are widely used in public and residential buildings because of their quick drying, lack of undesirable odor, good washability, excellent finish, and easy application. However, most of the synthetic insecticides are not water soluble, therefore a one pack composition where the insecticide is incorporated into the paint during manufacturing and is stable for many months is desirable. At present, Inesfly^®^ Paint 5A IGR NG, a water-based insecticidal paint from Inesfly Corporation, is a single pack system in which insecticide and insect growth regulator are mixed in an emulsified form and homogeneous mixing is required before application (1, 2). However, to the best of our knowledge, encapsulation of DM in polymeric binder particles at the time of polymerization has not been reported. Incorporating DM at the time of polymerization has many advantages, including that the insecticide will remain uniform, stable, and its slow and sustained release will be ensured for a wide period of time due to encapsulation in polymeric binder particles. This binder formulation is used to prepare slow-release insecticidal paint. With an objective to develop single pack water-based slow-release insecticidal paint formulation, we synthesized a series of latex binders with varied monomer and DM compositions at the time of polymerization. Binder composition suitable for paint formulation was selected on the basis of lower coagulum formation during polymerization, higher solid content (%), higher intrinsic viscosity, and higher DM content in binder emulsion.

Many studies have shown that the insecticide embedded in the matrix of different surfaces can produce substantial efficacy against a variety of arthropods vectors both in laboratory and field conditions. It was found that AIs loaded into the matrix are released slowly onto the surface and hence sustain for a longer time to provide consistent efficacy. Maloney et al. (5) has reported that insecticide embedded in the matrix provided more efficacy as compared to the insecticide alone after 9 months of application. Similarly, Yadav et al. (6) has shown that tarsal exposure to insect growth regulator (IGR) labeled surface for a brief period of time and at very low concentrations drastically influenced the fecundity, fertility, and adult emergence in wild *Ae. aegypti* mosquitoes.

In the pre-text of increased geographical expansion of vectors and extraordinary transmission of arthropod vector-borne disease in new areas, the idea of using insecticidal paint-based formulations is popular. Therefore, an attempt has been made to develop and optimize an effective insecticide-based paint formulation and to establish its residual activity by evaluating against a well-known mosquito vector.

## Methods

### Chemicals and Insecticide

Vinyl acetate (VA), methylmethacrylate (MMA), ethylacrylate (EA), methacrylic acid (MAA), and acrylic acid (AA) (all from Aldrich) were used as monomers. Sodium lauryl sulfate (SLS, Merck) and Triton X-100 (Aldrich) were used as emulsifiers. Ammonium persulfate (APS, Analytical grade, BDH) was used as free radical initiator. Technical grade deltamethrin (98% pure) obtained from M/S Tagros Chemical India, Chennai was used for encapsulation.

### Emulsion Polymerization

Emulsion polymerization was carried out using a previously reported method with some modifications (7). Polymerization was performed in a 1 L capacity three necked glass reactor equipped with an overhead mechanical stirrer, a reflux condenser, a thermometer, and two dropping funnels through a Y-shaped connector. Copolymers of varying composition were synthesized by semi-continuous emulsion polymerization technique. Initially, the reactor was charged with 200 g de-ionized (DI) water, 2 g SLS, and 5 g Triton-X. The reactor was heated to 75°C in a water bath and the rotation of the mechanical stirrer was adjusted to 100 rpm. All the monomers were weighted and mixed before feeding to the reactor (Table 1). Initiator solution was prepared with 0.2 g APS in 50 g DI water. When desired temperature was achieved, 5 ml of initiator solution was added to the reactor in a single shot. Monomer mixture was fed drop wise (150 ml in 135 min) in parallel with initiator solution (40 ml in 135 min). Subsequently, 5 ml of initiator solution was added in a single shot after the monomer feeding was completed. The polymerization was continued for another 2 h at the same temperature to achieve maximum conversion. Then the reactor was cooled to room temperature and polymer emulsion was filtered through cheesecloth and stored in high density polyethylene (HDPE) containers at room temperature.

**Table 1:** Monomer composition, solid content, and intrinsic viscosity of the binders.

**Code**	**Monomers**										**Solid content in binder emulsion(% w)**	**Conversion (%)**	**η_int_ (dL/g)**	**Acid content (mM)**
	**VA**		**EA**		**MMA**		**MAA**		**AA**					
	**w%**	**mol%**	**w%**	**mol%**	**w%**	**Mol%**	**w%**	**mol%**	**w%**	**mol%**				
P1	80	82.37	20	17.69	–	–	–	–	–	–	31	72	0.563	–
P2	60	63.66	40	36.33	–	–	–	–	–	–	33	77	0.860	–
P3	50	53.87	50	46.12	–	–	–	–	–	–	34	79	0.947	–
P4	40	43.77	60	56.22	–	–	–	–	–	–	34	79	1.024	–
P5	20	22.60	80	77.39	–	–	–	–	–	–	35	81	0.586	–
P6	–	–	100	100	–	–	–	–	–	–	33	77	0.408	–
P7	55	58.35	40	36.33	–	–	5	5.30	–	–	35	81	1.194	1.9
P8	50	53.05	40	36.33	–	–	10	10.61	–	–	25	58	1.246	2.1
P9	45	48.11	45	41.19	–	–	10	10.69	–	–	36	84	1.158	5.4
P10	25	27.99	50	47.94	25	24.06	–	–	–	–	37	86	1.572	–
P11	24.5	27.14	49.2	46.86	24.5	23.33	–	–	2	2.65	36	84	2.194	0.8
P12	23.5	25.68	48.4	45.67	23.5	22.06	–	–	5	6.58	40	93	2.563	2.5
P13	22.5	24.73	22.5	21.17	48	44.95	–	–	7	9.14	33	77	1.146	4.5
P14	21.5	23.41	21.5	20.04	47	43.59	–	–	10	12.94	35	81	0.863	6.3

*% w, % weight; η_int_, Intrinsic viscosity*.

### Microencapsulation of Deltamethrin

For DM microencapsulation, 200 g DI water mixed with 2 g SDS (Sodium-Dodecyl-Sulfate) and 5 g of Triton-X (non-ionic surfactant) was taken in a polymerization reactor. The temperature was raised to 75°C with continuous agitation. Monomer mixture was prepared with 72.6 g EA, 34.95 g MMA, and 34.95 g VA, and 7.5 g AA. DM (1–5 g) was added to the monomer mixture and stirred to dissolve it completely. Monomer mixture containing DM was fed slowly into the reactor over a duration of 135 min. Simultaneously 0.2 g of APS in 50 ml water was fed in for the same duration of time. The reaction was held for another 2 h at 75°C for complete conversion. Then the polymer emulsion was cooled to room temperature and filtered through cheesecloth and stored in HDPE containers at room temperature.

### Polymer Characterization

Polymer emulsion was broken by addition of 15% sodium chloride (NaCl) solution. The coagulated polymers were washed several times with distilled water and dried at 70°C for 24 h. Solid content of each emulsion was determined from the dry weight of coagulum and reported in percentage. Characterization of the dried polymers was carried out to determine their intrinsic viscosity (η_int_) and acid value. The η_int_ of the copolymers was determined by Ubbelohde viscometer in a thermostat at 30 ± 1°C. The acid content in the copolymers was determined by acid-base titration of 0.5% polymer solution in methanol with standard sodium hydroxide (NaOH) solution, in the presence of phenolphthalein as indicator.

### Thermal Studies

Differential scanning calorimetry (DSC) of the copolymers was carried out on DSC 1 STAR^e^ system (Mettler Toledo, USA) in nitrogen atmosphere from −40 to 200°C at a heating rate of 10°C/min. The sample size was between 4 and 5 mg in all the experiments. Thermo gravimetric analysis (TGA) was carried out on Pyris 1 TGA (Perkin Elmer, USA) in nitrogen atmosphere from room temperature to 600°C. The rate of heating was 20°C/min.

### HPLC Quantification

Binder samples were applied on glass panels and dried for 72 h at ambient condition. Approximately 0.5 g of the dried binder sample was taken in a 100 ml beaker. To extract DM, 25 ml of HPLC grade acetonitrile was added and sonicated for 5 min. After filtration the binder sample was extracted again with 25 ml acetonitrile. Filtrates were combined and transferred in to a 50 ml volumetric flask, and volume was made up by acetonitrile. One ml of the solution was filtered through 0.22 micron syringe filter (Millipore Inc., USA) and analyzed by HPLC. HPLC system (Waters, USA) equipped with 1,525 binary pump, 2,487 tunable dual wavelength UV detector, and Rheodyne injector with 10 μl loop was used in the present study. Analysis was performed by isocratic elution (methanol and water 8:2 v/v) on XTerra MS C18 (4.6 × 250 mm, 5 μm) reverse phase HPLC column (Waters, USA). Wavelength and flow rate were set at 280 nm and 1 ml/min, respectively. Standard solution was prepared by dissolving 14.78 mg of DM in 20 ml of acetonitrile. DM content was determined by comparing the corresponding peak area of sample with standard.

### ESEM-EDX Characterization

Different binders were characterized by Quanta 400 environmental scanning electron microscope equipped with energy dispersive X-ray analyzer (ESEM-EDX) for their microscopic homogeneity and phase formation morphology. Small round cover slips were mounted on brass SEM stubs with double-sided adhesive tape. Small drops of binder emulsion were placed on the cover slip and dried under vacuum at room temperature and then coated with gold in a JFC-1100 sputter coating unit. These specimens were than analyzed by ESEM for morphology. Micrographs were taken at different magnifications to ascertain phase homogeneity and micro-distribution.

### Preparation of Insecticidal Paint

Titanium dioxide (TiO_2_), Triton X-100, and SDS were mixed in requisite quantities with DM encapsulated binder thoroughly to prepare the emulsion paint. The emulsion paint was applied on a cement surface by brush for testing of the insecticidal property of the paint. Binder without DM was used to prepare paint as control. The paint compositions were prepared using different essential components such as binder, pigment (TiO_2_), and surfactants. However, the number of components other than binder was kept unaltered for all compositions to avoid any ambiguity in insecticidal properties.

### Bio-Efficacy Evaluation

Developed insecticidal paint at the rate of 8 square meters per liter (2.82 g of ready-to-use paint) was applied uniformly on 15 × 15 cm^2^ of cement surface and left to dry at ambient temperature for 24 h. After complete drying, anti-insect activity was determined using laboratory-reared *Ae. aegypti* mosquitoes following standard method as recommended by WHO (8). In brief, 4-to-5 day-old unfed female mosquitoes (*N* ≥ 41 for each time period; ≥3 mosquitoes/replicate) were exposed to the treated surface in a WHO cone bioassay (obtained from Universiti Sains Malaysia) for 30 min; thereafter the mosquitoes were transferred into the holding cups (150 ml capacity) (1). Mosquito knockdown was recorded after five min intervals up to 60 min, whereas the mortality was observed after 24 h of exposure. The mortality was corrected by taking into account the control mortality. Test females were left at a temperature of 27 ± 2°C and a relative humidity (RH) of 80% for 24 h delayed mortality assessments. During the holding period, 10% sugar solution was provided for feeding. The painted surfaces were stored carefully in aluminum foil at room temperature and similar experiments were performed again after 6 (T6), 12 (T12), and 18 months (T18) to determine the residual efficacy.

### Data Analysis

The mortality obtained in the mosquito species was corrected using Schneider-Orelli's formula (9). Knockdown time (KDT) along with slope and 95% confidence interval (CI) was determined using Log dose probit (Ldp) Line computer program. Chi-square (χ^2^) test was used to check the fitment of probit, whereas the linearity of data was evaluated using linear regression. Dunnett's multiple comparisons test has been used to compare the corrected mortality at different time intervals.

## Results

### Polymer Characterization

Solid content of an emulsion polymer indicates the actual content of polymer in the emulsion. The conversion of monomer to polymer can be calculated from actual polymer content with respect to theoretically maximum achievable polymer content, where all monomers are converted to polymers. Intrinsic viscosity (η_int_) indicates the molecular weight of the polymer, hence longer polymer chains are formed with higher value of η_int_, Furthermore, for binder application both percent conversion and η_int_ should be optimum to achieve the desired performance. Conversion of monomer to polymer depends upon the nature of monomers and reaction conditions. Copolymers of VA and EA (P1–P5) showed conversion between 72 and 81% and η_int_ between 0.563 and 1.024 dL/g. EA has a significant effect on both conversion and η_int_ of the resultant copolymers (P1–P5). Both the parameters displayed their optimum between 40 and 20 w% of EA in the monomer feed (Table 1). However, polyethylacrylate (P6) showed lower η_int_ than the VA-EA copolymers, whereas the introduction of MAA (P7–P9) marginally improved the η_int_ value. Nevertheless, the conversion for P8 was found to be 58% only. This may be due to lower EA than VA content in the monomer feed. When the feed was balanced with equal EA and VA content, 84% conversion was observed (P9), but our target for higher η_int_ was not achieved.

MAA is highly water soluble and weighs 10% of the total monomer in P8. Since EA is more hydrophobic than VA, the emulsification of MAA is enhanced by EA. Furthermore, at lower EA content in P8, the emulsification of MAA was less effective; therefore a significant amount of MAA was polymerized outside the micelles forming lumps in the reaction vessel. Hence the overall yield was low in the case of P8. Again, the acid content in P8 was also found to be low due to lumps formation. Extra-micellar polymerization of MAA was prevented and percentage conversion was increased when EA content was increased, as is evident for P9 (Table 1). MMA was introduced as a co-monomer and significant improvement was observed for P10 (η_int_ = 1.572 dL/g) with a good conversion of 86%. Introduction of AA as co-monomer (P11–P14) further improved η_int_ and showed an optimum value at 5 w% (P12) in the monomer composition. Acid content in polymeric binder was found to be increased with an increase in corresponding acid monomer feed from P11 to P14. It signifies that the introduction of hydrophobic MMA co-monomer prevented extra-micellar polymerization of the acid co-monomer during polymerization.

We started the synthesis of binders' (P1–P6) composition with VA and EA. Thereafter MAA was incorporated to increase the intrinsic viscosity of binders (P7–P9). Then P10–P14 MAA was replaced with MMA and AA in order to get higher intrinsic viscosity. Among the 14 binder emulsions synthesized, P12 showed optimum conversion and η_int_, and hence was considered for microencapsulation of DM.

### Deltamethrin Microencapsulation

**Figure 1:**
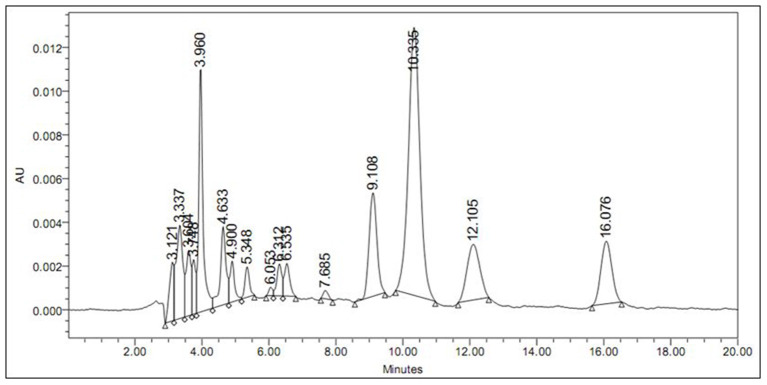
HPLC chromatogram of binder (PS001) extract containing DM (Rt = 16.076 min).

A total of four binders were synthesized encapsulating DM ranging between 0.68 and 3.2 w% in the monomer feed (Table 2). DM was completely soluble in the monomer mixture. During polymerization the parameters, such as temperature, stirring speed, and monomer feed rate were the same for all DM encapsulated binders. The actual concentration of DM in binder film was estimated by HPLC. The retention time (Rt) of DM recorded at 16.076 min was free from other interferences (Figure 1). Hence the described method was found to be suitable to estimate DM content in all the binders. Microencapsulation of DM first increased and then decreased with increase in DM concentration in monomer feed. This may be attributed to the coagulum formation. The amount of coagulum was found to be increased with an increase in DM content in the monomer feed, as evident in Table 2. Increase in DM concentration in the feed caused extra-micellar polymerization, which contributed to the coagulum formation. Furthermore, the loss of DM was also found to be increased due to coagulum formation as evident from Table 2. Therefore, incorporation of DM in the binder has an upper limit as the entire batch is coagulating at 3.2 w% of DM content. Binder attained an optimum DM content when monomer feed contained 1.3 w% of DM, as shown in Table 2. Therefore, PS002 (the binder with optimum DM content) was used to prepare the paint formulations (Table 3). These formulations were used for preparing painted surfaces for evaluating the anti-insect activity.

**Table 2:** Theoretical and estimated deltamethrin (DM) content in binder emulsion and glass transition temperature (Tg).

**Polymer code**	**Solid content (w%)**	**Conversion (%)**	**DM in monomer feed (w%)**	DM content in binder (w%)^*^	**^#^Tg (°C)**
P12	40	93	0	0	15.1
PS001	39	91	0.68	0.95	8.09
PS002	38	88	1.3	1.18	10.1
PS003	34	79	2.2	1.09	10.5
PS004	–	–	3.2	–	9.91

* *Determined by HPLC;*

# *Tg, Glass Transition temperature; DM, deltamethrin*.

**Table 3:** Composition of paint formulations developed in the study.

**Code**	**PS 002 (g)**	**Rutile TiO_**2**_ (g)**	**Triton-X (g)**	**SDS (g)**	**Water (g)**	**25% Ammonia (g)**	**DM content w%^#^**
WSRIP-1	10	10	2.5	2.5	10	0.5	0.24
WSRIP-2	15	10	2.5	2.5	10	0.5	0.44
WSRIP-3	20	10	2.5	2.5	5	0.5	0.59
Control	20^*^	10	2.5	2.5	5	0.5	0

* *Binder P12 was used in place of PS002*,

# *calculated*.

### Thermal Analysis

**Figure 2:**
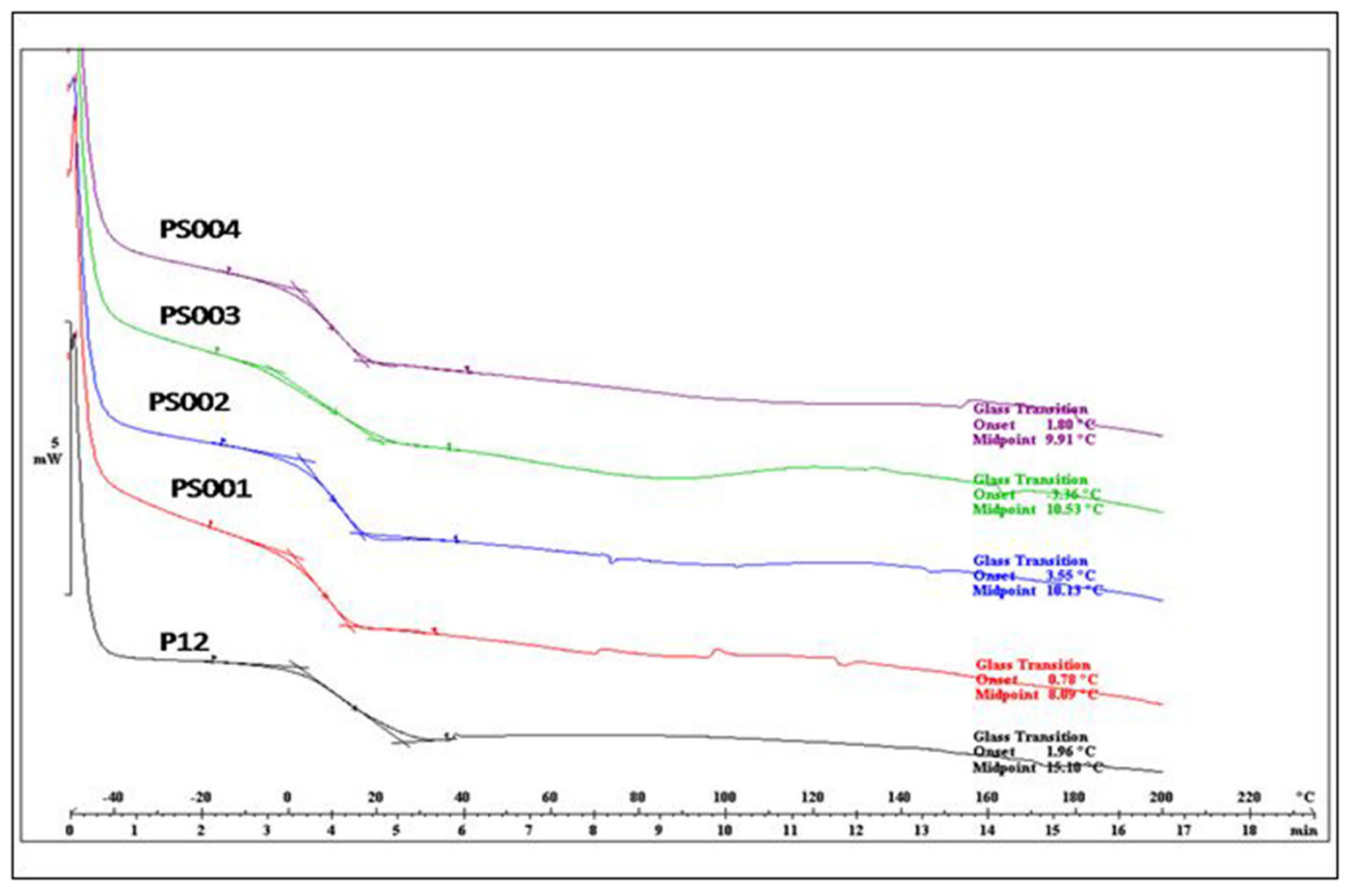
Glass transition temperature (Tg) of binders with different concentrations of DM; P12 (0%), PS001 (0.68%), PS002 (1.3 %), PS003 (2.2%), PS004 (3.2%).

**Figure 3:**
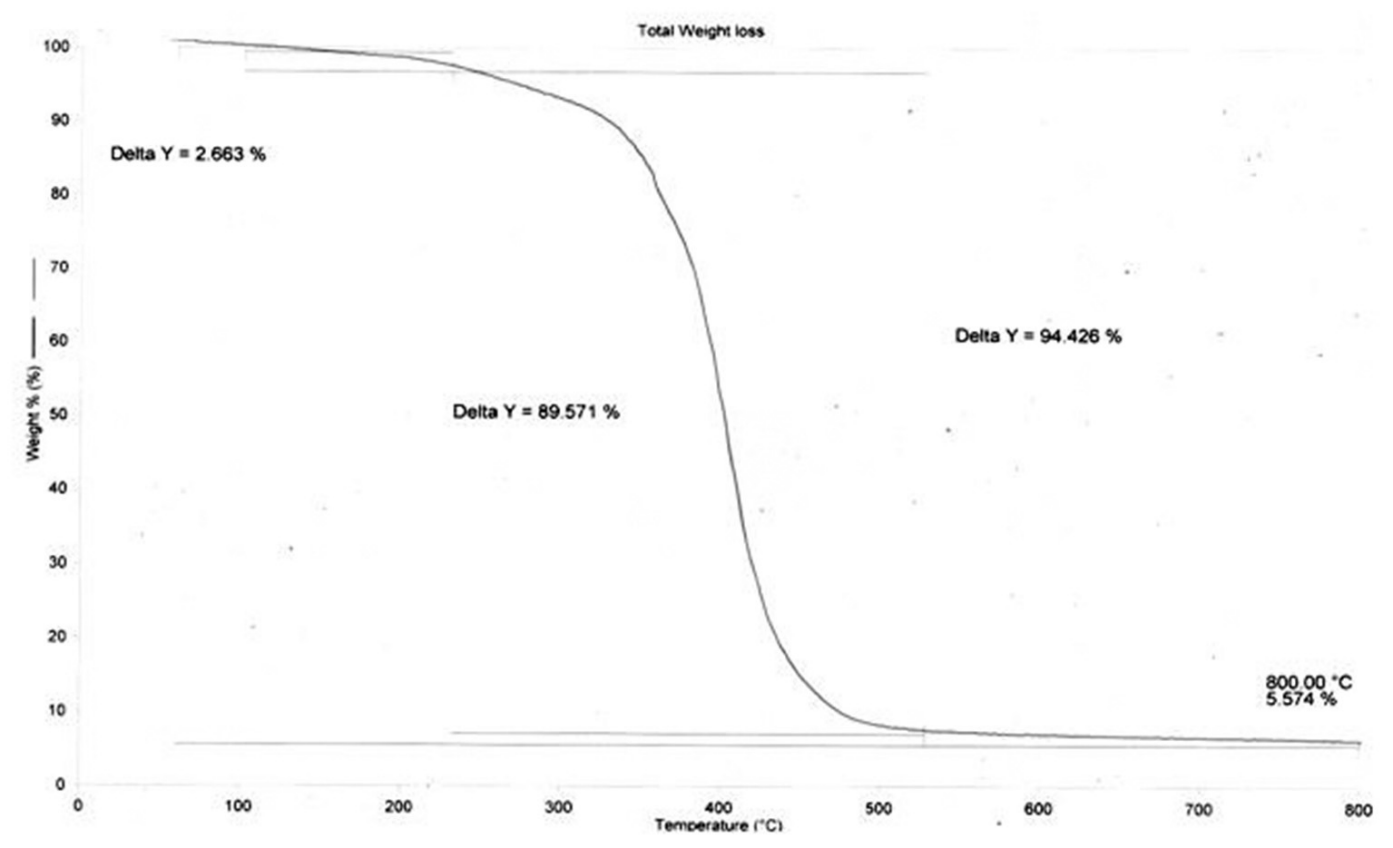
Thermo gravimetric plot of binder PS002.

Glass transition temperature (T_g_) of all the binders were determined by differential scanning calorimetry (DSC). T_g_ of all the DM encapsulated binders were found to range from 8 to 11°C (Figure 2), however T_g_ of binder without DM (P12) was recorded at 15.1°C. It is evident from Table 2 that DM is modifying the T_g_ of the polymer matrix, however to a lesser extent, and does not have a considerable plasticizing effect. Degradation of both with DM and without DM binders followed a similar pattern in TGA. A sharp decrease in mass was observed at 400°C (Figure 3) which is typical of acrylic copolymers (5).

### ESEM-EDX Characterization

**Figure 4:**
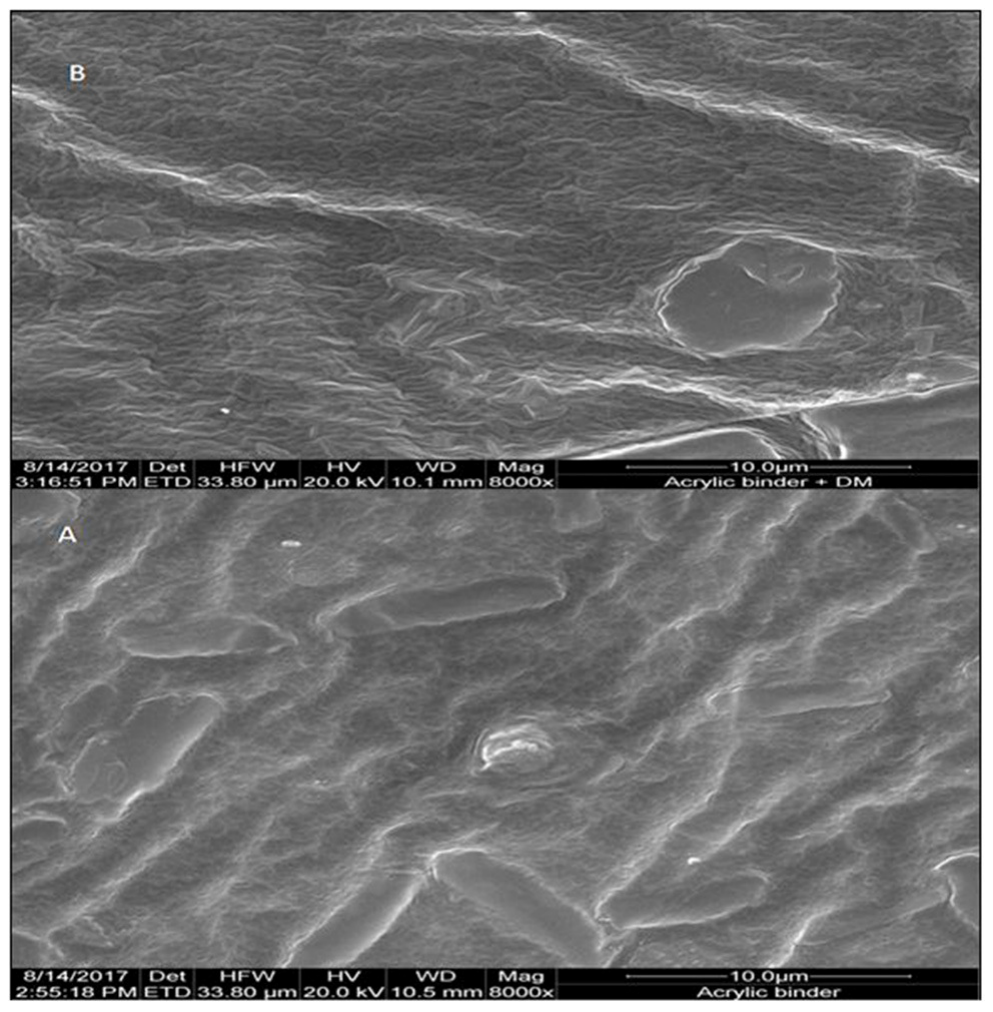
**E**SEM micrographs of binders without deltamethrin P12 **(A)** and with deltamethrin PS002 **(B)**.

**E**SEM pictures of binder without DM (P12) and with DM (PS002) are represented in Figures 4A,B, respectively, at 10 μm resolution. Cracks were observed on both P12 and PS002 binders and the surfaces were not smooth. Some grain-like micro crystals of DM and surfactant SLS were found uniformly distributed on the PS002 surface. Such structures were absent on the surface of P12.

### Effectiveness of Insecticidal Paint Against Dengue Vector *Ae. aegypti*

The paint formulations as given in Table 3 were evaluated against lab-reared and maintained *Ae. aegypti* mosquitoes in the laboratory. Among the three formulations, WSRIP-3 was found to retain the highest DM content (0.59 w%) as compared to the others. Therefore, this formulation was evaluated for up to 18 months at 6-month intervals. It was found that the DM contents (%) were 0.31, 0.24, and 0.19% after 6 (T6), 12 (T12), and 18 months (T18) of evaluation, respectively.

**Figure 5:**
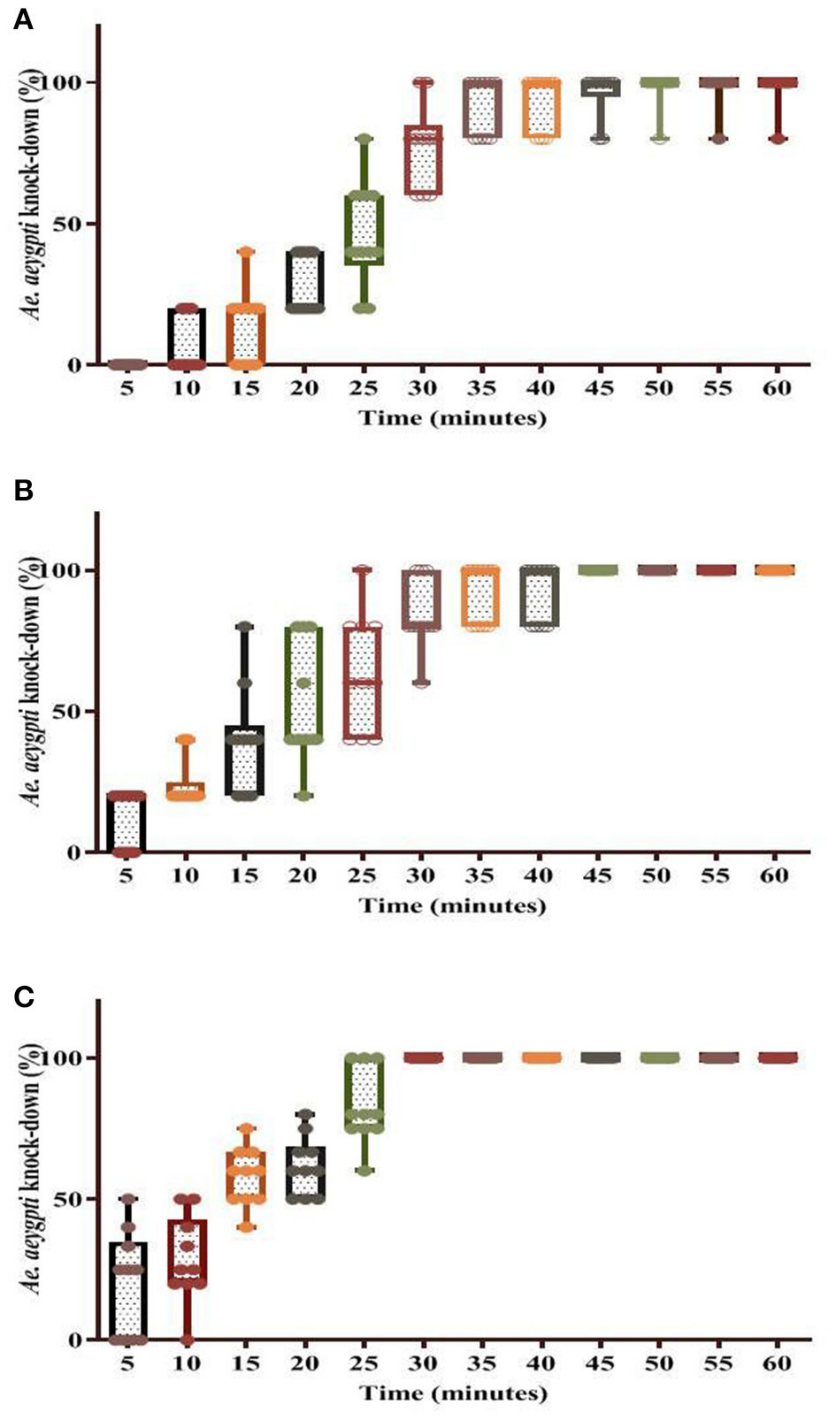
Knockdown rate of dengue vector *Ae. aegypti* using different formulations at time T0. **(A)** WSRIP-1; **(B)** WSRIP-2; **(C)** WSRIP-3.

**Figure 6:**
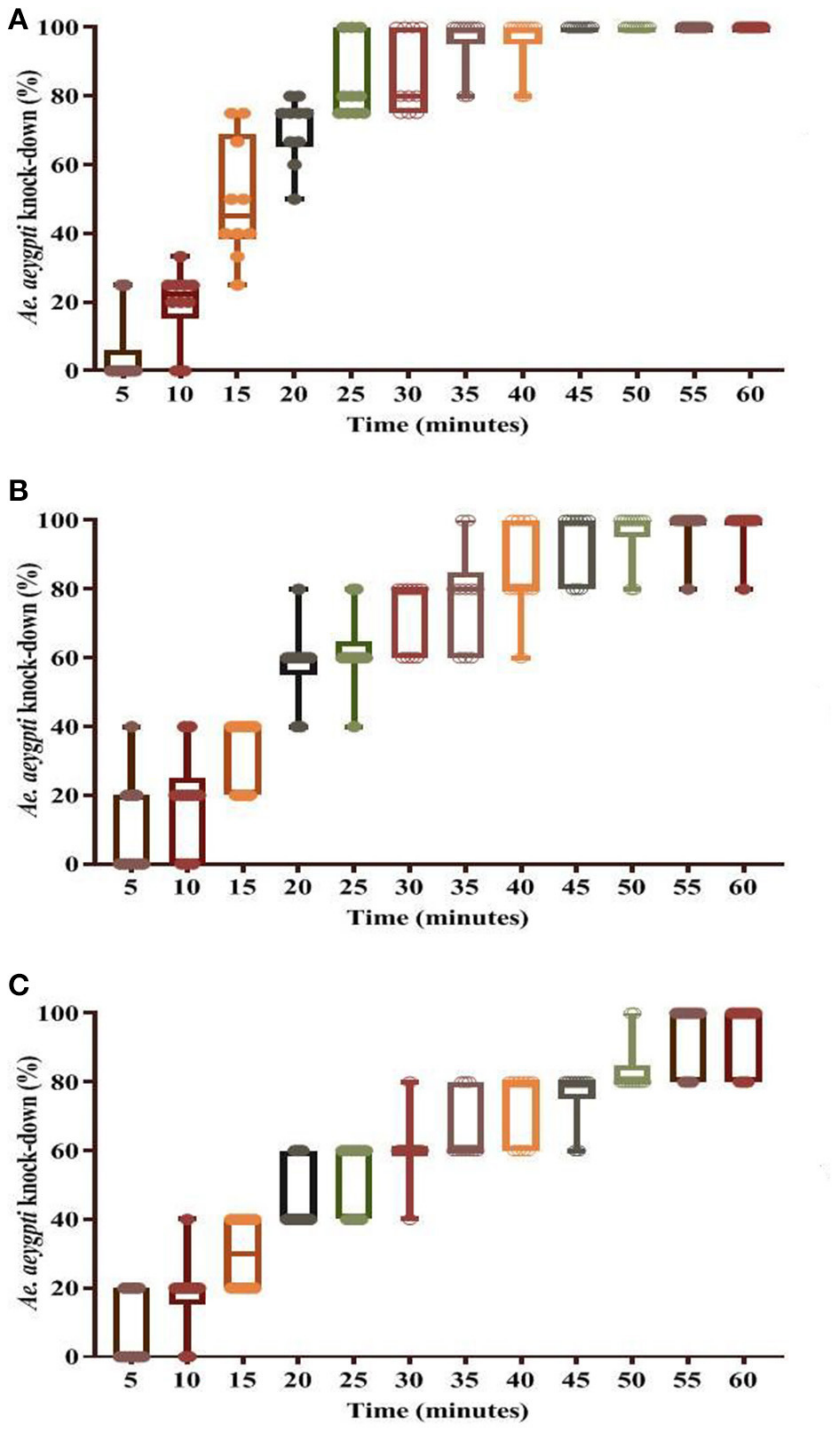
Knockdown rate in dengue vector *Ae. aegypti* using insecticidal paint formulation (WSRIP-3). **(A)** after 6 months (T6); **(B)** after 12 months (T12); **(C)** after 18 months (T18).

The percentage knockdown observed for the tested *Ae. aegypti* mosquitoes has been shown in Figures 5, 6. It was found that at T0 (freshly applied formulation) the KD (%) was 98% in WSRIP-1, while it was 100% in WSRIP-2 and WSRIP-3 formulations, respectively, post 60 min of exposure (Figure 5). The corrected mortality (post 24 h) was also found to be 100% in all the three formulations at T0. Probit model suggested that the tested formulations showed KDT_10_ values ranging from 4.0 to 13.7 min, with KDT_50_ values ranging from 13.4 to 23.1 min at T0 (Table 4). Although mortality was similar in all three formulations at T0, WSRIP-3 formulation was still more effective as compared to the other formulations as the KDT_10_ and KDT_50_ values for this formulation were 4.0 min (95% CI: 2.2–5.6) and 13.4 min (95% CI: 11.2–16.0) respectively. Considering the uptake of DM and T0 bio-efficacy, WSRIP-3 was taken for long-term efficacy evaluation.

**Table 4:** Knockdown and 24 h delayed mortality in susceptible *Ae. aegypti* for different formulations.

**Time interval**	**Formulation** **code**	**Deltamethrin** **content w%**	**KD_**60min**_ % (*N*)**	**KDT_**10**_ (95% CI)**	**KDT_**50**_ (95% CI)**	**Slope (±)**	**χ^**2**^ (*p*)**	***R***	**CM_**24 h**_ (%)**
T0	WSRIP-1	0.24**^#^**	98 (50)	13.7 (12.1–15.1)	23.1 (21.6–24.6)	5.6 ± 0.4	10.9 (0.3)	0.98	100
	WSRIP-2	0.44**^#^**	100 (50)	6.1 (4.6–7.6)	16.2 (14.3–18.0)	3.1 ± 0.3	10.7 (0.1)	0.96	100
	WSRIP-3	0.59**^#^**	100 (42)	4.0 (2.2–5.6)	13.4 (11.2–16.0)	2.8 ± 0.4	3.5 (0.3)	0.96	100
T6	WSRIP-3	0.31^&^	100 (41)	6.8 (5.2–8.3)	15.2 (13.4–16.9)	3.7 ± 0.4	5.4 (0.5)	0.99	97.6
T12	WSRIP 3	0.24^&^	98 (50)	6.9 (5.4–8.4)	18.1 (16.2–19.9)	3.1 ± 0.2	12.9 (0.2)	0.97	94
T18	WSRIP 3	0.19^&^	94 (50)	7.4 (5.6–9.1)	22.1 (19.7–24.3)	2.7 ± 0.2	8.6 (0.6)	0.97	90

*T0, at the time of application; T6/T12/T18, 6/12/18 months post-application; CM_24 h_, Corrected mortality post 24 h exposure; KDT, knockdown time; CM_24 h_, 24 h corrected mortality;*

# *calculated*,

& *estimated by HPLC; p significant if < 0.05*.

Bio-efficacy results suggested that KD in WSRIP-3 did not decline and remained at 100%, however the corrected mortality declined non-significantly to 97.6% (95% CI: 93.5–102.5) (*p* = 0.9) at T6 (Table 4). Nevertheless, there was an increase in the KD_10_ and KD_50_ values to 6.8 min (95% CI: 5.2–8.3) and 15.2 min (95% CI: 13.4–16.9) respectively (Figure 6). At T12, percent KD and corrected mortality were found to be reduced to 98% (KDT_50_ = 18.1 min) and 94% (95% CI: 87.1–100.9) (compared to T0, *p* = 0.2) respectively. Furthermore, KDT_10_ and KDT_50_ values were 7.4 min (95% CI: 5.6–9.1) and 22.1 min (95% CI: 19.7–24.3) respectively, whereas 24 h corrected mortality was found to be 90% (95% CI: 82.5–97.5) (compared to T0, *p* = 0.02) after T18. The probit model used to determine KDT values did not deviate from the linearity and displayed normal distribution of percent knockdown with time for the tested formulations at different time intervals (*p* ≥ 0.1).

## Discussion

In the past few years, a variety of insecticidal paints have become available commercially for achieving protection from hematophagous arthropod vectors (1, 2, 5). However, their availability and use in households was primarily restricted to USA and some European countries, where such formulations were promoted against arthropod vectors that tend to feed indoors and mainly dwell on the walls and ceilings of human houses (10). The concept, although promising, did not gain much popularity in the majority of developing countries compared to the existing and less costly interventions, such as Indoor Residual Spray (IRS), which is effective but may not provide consistent efficacy for a longer time. At present, advances in paint technology have enabled researchers to guide insecticidal paint formulations to have insecticide or a mixture of insecticides embedded into the matrix which releases slowly on to the dried paint surface (10). Studies have shown that IRS is not considered to control adult *Ae. aegypti* except during outbreaks (11), therefore suitable paint formulations may provide consistent efficacy against *Aedes* vectors and other desired insects for a considerably longer time.

Presently, different compositions of emulsion binder using monomer and DM were prepared and the best optimized composition was formulated into insecticidal paint to test against known dengue vector. It is well-known that surface coating quality is a critical element in any paint formulation. Surface coating is a process where a liquid material is spread over a surface and forms a thin film. It is used for protection, aesthetic attraction, and some other functional purposes like protection from insects (12). The surface coatings, in general, are made up of four basic components: binder resin, pigments, solvent, and other additives. The main part of coating is binder resin which is the film-forming agent of the coating material. In recent years, acceptability to the water-based emulsion paints has increased, primarily due to their low cost, stability, quick drying, and quick recoatability (13). Polymer VA is widely used in the production of emulsion binder of water-based emulsion paints (14). However, the Tg of polyvinyl acetate (PVA) is 29°C, therefore it is often copolymerized with EA to obtain a softer composition for use as emulsion (15). Since lower acrylate polymers like EA have Tg below room temperature, they are typically soft and rubbery, hence a small amount of MMA was added to impart strength and hardness to the polymer film (16). A small amount of acid monomer (AA and MAA) was also used to provide adhesion and thermosetting capability to the polymer film.

Monomer composition has a significant effect on polymerization and end properties of the binder emulsion (17). Higher solid content and larger polymer molecules contribute significantly to the film forming properties of the binder emulsion. Therefore, different compositions (Table 1) of acrylic monomers have been used to prepare the binder emulsion in the present study. Conversion of monomer to polymer is crucial in polymerization, as the monomer not converted to polymer will remain in the binder emulsion and may adversely affect the film property (18). In the present study, the theoretical solid content of all the emulsion would have been 43% under complete conversion of monomer to polymer. Conversion of monomer to polymer was determined from percent solid content of the emulsion after polymerization with respect to theoretical solid content. However, a small part of the polymer is also lost due to formation of coagulum. Strength of the binder film depends upon the molecular weight of the polymer molecules. The file strength tends to become better with the increase in the molecular weight. Polymer η_int_ has been regarded as an indicator of its molecular weight, which increases with the increase in the molecular weight. Hence, along with the percent solid content, η_int_ was also considered as a parameter to optimize monomer composition of emulsion binder (Table 1).

The estimated DM contents at different time intervals were suggestive that the developed formulation has a slow-release mechanism which enables the DM to gradually release to the surface. The slow-release mechanism ensures the availability of DM on surface and hence provides considerable residual effectiveness even after 18 months of application (Table 4). It has been shown previously that such formulations can offer protection against a variety of arthropod vectors that play important roles in transmitting various diseases such as malaria, dengue, filaria, Zika, chikungunya, leishmaniasis, and chagas disease (10). Present results have shown that the insecticidal paint formulation provided 18 months residual efficacy by producing 94% knockdown and 90% mortality against a well-known dengue vector of the Indian region. Amelotti et al. (19) have evaluated organophosphate (Inesfly^®^ 5A IGR™) and a pyrethroid-based insecticidal paint (Inesfly^®^ 5A IGR NG™) formulation against *Triatoma infestans* and reported that pyrethroid formulations showed 84% while organophosphate formulation displayed 98% mortality after 12 months of the application on different surfaces (19). Similarly, Mosqueira et al. (1, 2) have shown that insecticidal paint Inesfly^®^ 5A IGR™ has been found to be effective against malaria vector *An. gambiae* in both laboratory and field conditions. It was reported that the mortality was 93–100% after 12 months of application in laboratory (1), while 90–100% against pyrethroid-resistant mosquitoes in experimental huts in the field (2). However, the efficacy after 12 months in the treated huts was found to have decreased to 60–80% (2).

The optimized formulation presently displayed encouraging efficacy and physiochemical properties, suggesting that the formulation has the potential to be evolved commercially after evaluation for a considerably longer time in different endemic settings. Many studies have argued that the slow release of insecticides from the paint layer could make the insecticide available on the surface or the surrounding surface for a considerable time, thereby providing protection for a longer duration compared to the traditionally encouraged interventions (8). The present formulation can be further improved by using different insecticides and insect growth regulators in optimized concentrations to form a single formulation, thereby offering a combination of insecticides for application against the target insect vectors. Similar formulations have the advantage that these can be applied indoors and outdoors by any individual without any special logistic planning.

The slow-release water-based insecticidal paint formulation developed presently was stable and produced high residual mortality against dengue vector in laboratory. Although we did not evaluate it for a long time, the achieved results after 18 months are sufficient to suggest that the formulation would be effective for a longer time against different vectors. The formulation after field evaluation could be an attractive tool to control hematophagous vector abundance in human houses and other peri-domestic structures with an advantage of embellishment, mainly in rural areas. However, in addition to monitoring the long-term human safety aspects and the effect on the environment, limitations associated with the use of this product also need to be acknowledged. The formulation may not perform well in remote settings where people live in mud-plastered porous houses. Although the formulation was tested for up to 18 months for efficacy under laboratory conditions, the actual impact of environmental factors, such as sunshine, rainfall, humidity, and wind speed on the formulation also needed to be studied in field trials to better understand its actual service life.

## Data Availability Statement

The original contributions presented in the study are included in the article/supplementary material, further inquiries can be directed to the corresponding author.

## Author Contributions

BA and SD conceptualized and designed the study. RA and BA performed the development and physico-chemical evaluation of the formulation. SD and KY maintained the *Ae. aegypti* mosquitoes and conducted the anti-mosquito study. PP performed the microscopy study. SD and BA analyzed the data. BA and SD drafted, while DS edited the manuscript. All authors read and approved the manuscript.

## Conflict of Interest

The authors declare that the research was conducted in the absence of any commercial or financial relationships that could be construed as a potential conflict of interest.

## Publisher's Note

All claims expressed in this article are solely those of the authors and do not necessarily represent those of their affiliated organizations, or those of the publisher, the editors and the reviewers. Any product that may be evaluated in this article, or claim that may be made by its manufacturer, is not guaranteed or endorsed by the publisher.
